# Eleven-month-old infants infer differences in the hardness of object surfaces from observation of penetration events

**DOI:** 10.3389/fpsyg.2015.01005

**Published:** 2015-08-03

**Authors:** Tomoko Imura, Tomohiro Masuda, Nobu Shirai, Yuji Wada

**Affiliations:** ^1^Department of Information Systems, Faculty of Information Culture, Niigata University of International and Information Studies, Niigata, Japan; ^2^Faculty of Human Sciences, Bunkyo University, Koshigaya, Japan; ^3^National Food Research Institute, National Agriculture and Food Research Organization, Tsukuba, Japan; ^4^Department of Psychology, Niigata University, Niigata, Japan

**Keywords:** infant vision, material perception, motion perception, perceived hardness of solid objects, object manipulation

## Abstract

Previous studies have shown different developmental trajectories for object recognition of solid and non-solid objects. However, there is no evidence as to whether infants have expectations regarding certain attributes of objects, such as surface hardness, in the absence of tactile information. In the present study, we examined infants’ perception of the hardness of object surfaces from visually presented penetration events using the familiarization–novelty preference procedure. Experiment 1 showed that by 11 months old infants distinguished a relatively soft surface from a crusty surface based on changes in the velocity of a moving object as the moving object penetrated the surface of the target object. Experiment 2 ruled out the possibility that infants were merely sensitive to differences in the velocity changes in the stimuli.

## Introduction

Basic physical properties such as the rigidity, elasticity, and viscosity of an object’s surface can influence patterns of locomotive behavior and object manipulation in adults and infants (Gibson and Walker, 1984; Gibson et al., 1987; Gibson and Pick, 2000). Adults use visual motion cues to predict the material properties of objects (Spelke, 1990; Doerschner et al., 2011; Masuda et al., 2011, 2013, 2015; Tani et al., 2013; Kawabe et al., 2015). For instance, visual cues regarding the deformation and penetration of object surfaces provide important information that allows us to distinguish solids from liquids (Spelke, 1990). Solids usually maintain their shape when they are moved, but liquids tend to deform as a result of the application of force from the outside. In addition, solids are less likely to be penetrated by other objects than are liquids.

Recent psychophysical research has shown that motion information enables us to distinguish not only whether an object is solid or liquid but also its material properties such as the hardness, elasticity, and viscosity of its surface (Masuda et al., 2011, 2013, 2015; Kawabe et al., 2015). We can also take into account the effects of motion parallax induced by our own head movements to judge the glossiness of object surfaces (Tani et al., 2013). However, no developmental studies of material perception have investigated how these mechanisms for distinguishing the material properties of objects are acquired by children. When do infants begin to distinguish solids from liquids and to identify the material properties of objects based on visual information?

Previous developmental studies investigating object permanence in infancy suggest different developmental trajectories for object recognition of solid and non-solid objects. Numerous studies have measured looking times in response to a violation of visual expectations; these studies suggest that infants between 2 and 12 months of age can represent several hidden objects in memory, even tracking the insertion or removal of objects behind a screen (Wynn, 1992; Koechlin et al., 1998; Aguiar and Baillargeon, 1999; Uller et al., 1999; Hespos and Baillargeon, 2001a,b; Feigenson et al., 2002). In contrast, several studies using non-solid objects demonstrated that 8-month-old infants failed to represent the number of non-solid or non-cohesive objects in similar experimental situations (Chiang and Wynn, 2000; Huntley-Fenner et al., 2002). A study by Huntley-Fenner et al. (2002) compared three different kinds of substances, solid and cohesive objects, non-solid and cohesive objects, and non-solid and non-cohesive objects (quantities of sand), and found that infants could retain representations of two solid and cohesive objects or two non-solid and cohesive objects hidden behind two different screens, but they failed to establish representations of two separate quantities of sand hidden behind two screens. The results suggest that cohesive objects, regardless of whether they are solid or non-solid objects, have a privileged status with respect to object representation, implying that the developmental progression of object recognition may differ depending on the object material.

On the other hand, a study using a habituation–dishabituation procedure found that 5-month-old infants were able to distinguish solids from liquids using deformation and penetration cues (Hespos et al., 2009). In that study, infants were habituated to an event in which an experimenter repeatedly tilted a cup containing either a liquid or a solid. Infants could observe whether the object in the cup moved in direct response to the cup’s inclination (liquid), or whether the object did not deform even when tilted (solid). On subsequent test trials, two kinds of events were shown to the infants: one in which the contents of a cup were poured into another cup (liquid), and another in which the contents of a cup were transferred into another cup without deformation (solid). Infants looked longer at events depicting a novel kind of material than at videos depicting a familiar one. In an additional experiment, infants were habituated to an event in which an experimenter tilted a cup containing a liquid or a solid in a similar manner to the first experiment, and they were subsequently tested with two kinds of events: one in which a stick pierced through the surface of an object (liquid), and another in which the same stick came to rest on top of an object (solid). Again, 5-month-old infants looked longer at a video depicting a novel kind of material than at a video of a familiar one. These findings suggest that 5-month-old infants are able to use deformation and penetration cues to distinguish liquids from solids.

Results have been inconsistent with respect to the developmental trajectory of material perception. The various discrepancies in experimental results may be due in part to differences in experimental procedures across studies. In the experiments reported by Huntley-Fenner et al. (2002), infants were required to identify objects hidden by screens and to establish and retain mental representations of these objects. It may have been difficult for 8-month-old infants to establish multiple representations of objects through time and space, even if the infants had the requisite sensitivity to distinguish solids from non-solid objects. Hence, it is necessary to use different stimuli and procedures to determine whether infants can indeed infer the material properties of objects from visual events. Additionally, most existing developmental studies have focused on infants’ ability to distinguish solids from non-solid objects. However, it is also important to investigate how infants perceive specific attributes of materials such as the hardness of object surfaces.

In the present study, we examined infants’ perception of the hardness of object surfaces based on visually presented penetration events. According to Masuda et al. (2011), adults can determine object surface hardness based on changes in penetration velocity. They showed participants videos in which a static hemisphere was penetrated by a stick, and asked them to judge the hardness of objects using an analog scale. The stimuli involved differences in stick velocity but no deformations of the hemispheric surface. The results of their experiments indicated that the surface of a penetrated object was perceived as harder when the velocity of the stick decreased just prior to, or at the beginning of, penetration. On the other hand, the surface of the penetrated object was perceived as less hard when the penetrating object was relatively faster after penetration than it was prior to penetration. These results suggest that adults can judge the hardness of a surface by changes in penetration velocity, even without deformation of the object surface.

We examined whether infants aged 7–12 months can infer hardness of object surfaces using changes in the velocity of a penetrating object as visually depicted in videos developed by Masuda et al. (2011). Because the stimuli were dynamic two-dimensional (2-D) computer graphics movies presented on a computer screen, the infants have to perceive three-dimensional (3-D) representation of stimuli from pictorial depth cues, such as occlusion and cast shadows. Accumulating developmental studies on depth perception suggest that infants perceive 3-D representations from 2-D images based on pictorial depth cues by 7 months (Kavšek et al., 2012). Thus, the current stimuli would not be suitable for testing infants aged <7 months.

## Experiment 1

### Materials and Methods

#### Participants

This study involved three groups of infants divided by age: 7–8 months (*N* = 18, nine males and 10 females, *M* = 230.8 days, SD = 20.0 days), 9–10 months (*N* = 19, 11 males and eight females, *M* = 294.4 days, SD = 17.3 days), and 11–12 months (*N* = 19, 10 males and nine females, *M* = 347.3 days, SD = 16.2 days). Two additional infants who participated in the experiment were excluded from the final sample due to excessive crying. Participants were recruited through leaflets distributed to the families of infants at the public health center and its branches in Niigata City. All parents of the participants provided informed consent. This study was approved by the Ethics Committee for Psychological Research, Niigata University, and was conducted according to the principles outlined in the Declaration of Helsinki.

#### Apparatus

Visual stimuli were presented on a 22′ cathode-ray tube (CRT) monitor (refresh rate: 60 Hz; resolution: 1024 × 768 pixels; color mode: 8 bit). Each infant watched the monitor while seated on a parent’s lap at a viewing distance of approximately 40 cm. To record the infant’s gaze direction, a small charge-coupled device (CCD) camera was attached below and at the center of the monitor. The CCD camera was connected to a separate TV monitor outside the experimental booth so that an experimenter could observe the infant’s viewing behavior through this monitor.

#### Visual Stimuli

**FIGURE 1:**
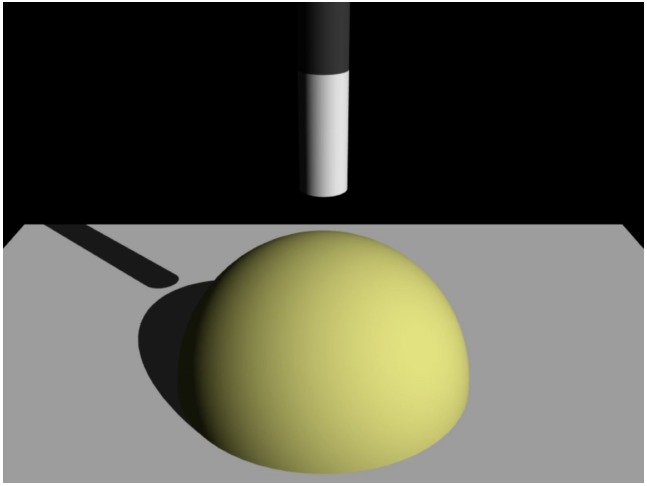
**Example of stimuli used in Experiment 1**.

**FIGURE 2:**
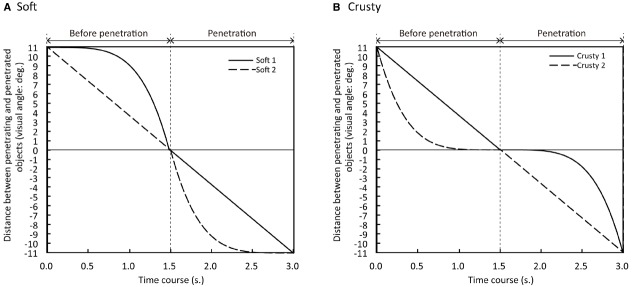
**Combinations of velocity changes before and during penetration employed in (A) soft and (B) crusty stimuli used in Experiments 1 and 2**.

We used stimuli developed by Masuda et al. (2011) to assess hardness perception in infants. The stimuli included the following visual elements: a striped stick, a yellow hemisphere, and a gray floor on a black background (Figure 1). The overall size of each stimulus was 44.8 × 33.6°, and stimuli were always presented at the center of the monitor. In each stimulus video, a stick approached and pierced the top of the hemisphere (corresponding to the “before penetration” phase), penetrated the hemispheric object, and came to rest on the floor (corresponding to the “penetration phase”). The distance traveled by the moving stick was 11.0°, and the duration of each phase in the event was 1.5 s; thus, in each video, an entire penetration event was 3 s in duration. The event was repeated three times in each trial, yielding a total duration of 9 s for each trial. We manipulated three velocity changes (deceleration, constant velocity, and acceleration) involving the stick’s velocity during the “before penetration” and “penetration” phases, creating two different types of stimuli in which the surfaces of the hemispheric objects were perceived by adult observers as relatively “soft” or “crusty” (Masuda et al., 2011). Two of the three velocity changes were combined. For one of the “soft” videos, the stick accelerated before penetration and then moved at a constant speed during penetration (Soft 1). The other “soft” video involved constant velocity of the stick before penetration and deceleration during penetration (Soft 2). In contrast, for one of the “crusty” stimuli, the stick approached at constant speed and then accelerated during penetration (Crusty 1). The other “crusty” video involved deceleration of the stick before penetration followed by constant velocity during penetration (Crusty 2). Examples of a preview version of the movies are shown on the website (http://dx.doi.org/10.6084/m9.figshare.1449011). The four combinations of the velocity changes are shown in Figure 2. The videos were created using 3D rendering software (Lightwave 9.0, D-storm).

The three velocity changes (deceleration, constant velocity, and acceleration) were defined as follows: The mode of the velocity change that occurred over the distance of the stick’s movement was given as a power function of time, as in the following equations, applied to both “before penetration” and “penetration” phases: Acceleration: Y = 11.0 × T^4.48^; Constant velocity: Y = 11.0 × T; and Deceleration: Y = 11.0 × [1.0–(1.0–T)^4.48^]. The parameter Y indicates the distance moved by the stick (ranging from 0.0 to 11.0° of visual angle). The parameter T indicates the time elapsed during the “before penetration” and “penetration” phases. This parameter ranged from 0.0 to 1.0 s for the movement of the penetrating object in the “before penetration” phase (both the moving distance and the moving duration were the same under the three velocity change patterns, whereas the initial and final velocity varied with each velocity change pattern; see also Figure 2).

#### Procedure

Each infant sat in a darkened room on a parent’s lap, approximately 40 cm from the screen. Parents were instructed not to look at the screen and not to interact with their infants during the experiment. Once infants looked at the center of the monitor prior to each trial, the stimulus video started playing. The infants were exposed to both of two experimental conditions: “crusty” and “soft.” Each experimental session consisted of four familiarization trials and two test trials. Each infant viewed one of the following videos during familiarization: “Soft 1” or “Soft 2” (soft condition) and “Crusty 1” or “Crusty 2” (crusty condition) for 9 s per trial. In subsequent test trials, two videos were presented, one at a time, for 9 s per trial. One of the two videos in the test phase was a novel stimulus in which the surface of the hemisphere appeared (to adult observers) to be of a different hardness than the hemisphere seen previously in the familiarization trials (novel trials). The other video in the test phase was a stimulus that consisted of velocity combinations identical to those of the first test stimulus, but in the opposite order, such that the surface of the hemisphere appeared to be of similar hardness to the familiarization trials (familiar trials). The test stimuli were presented sequentially, as infants could show looking preferences based on differences in local velocity between pairs of test stimuli (such as initial speed of a stick). Hence, while both test videos involved novel velocity changes, only the novel trials involved changes that were perceived as indicating different object surface qualities by adult observers. The four combinations of videos for familiarization and test trials are shown in Table 1. Their order of presentation between “soft” and “crusty” conditions was counterbalanced across infants.

**TABLE 1:** **The combinations of stimuli used in the familiarization and test trials for each condition**.

**Condition**	**Familiarizatíon**	**Test**
Soft	Soft l	Soft 2 vs. Crusty 2
	Soft 2	Soft 1 vs. Crusty 1
Crusty	Crusty 1	Soft 2 vs- Crusty 2
	Crusty 2	Soft 1 vs- Crusty 1

If infants, like adults, are able to discriminate the hardness of the hemispheres’ surfaces, they should look longer at test trials that depict a hemisphere of a different hardness than the object presented in the familiarization trials (novel trials) relative to test trials that appear to preserve the surface hardness of the hemisphere (familiar trials). On the other hand, if infants are merely sensitive to velocity changes involving the stick, rather than to attributes of object surfaces, then they should look at both stimuli for equal amounts of time, as both of the test videos include novel velocity changes.

An experimenter who was naïve to the identity of the stimuli assessed the infants’ gaze direction based on the video recording. The total time spent looking at a particular stimulus was calculated for each trial. Interobserver reliability was determined for the looking durations of each trial for 14 infants (*r* = 0.91). To determine whether infants looked longer at novel versus familiar trials in the test phase, we calculated preference scores corresponding to the proportion of the total looking spent looking at novel or familiar trials relative to the total looking time. We then performed *t*-tests for each age group to compare preference scores against a chance level of 0.5.

### Results and Discussion

**FIGURE 3:**
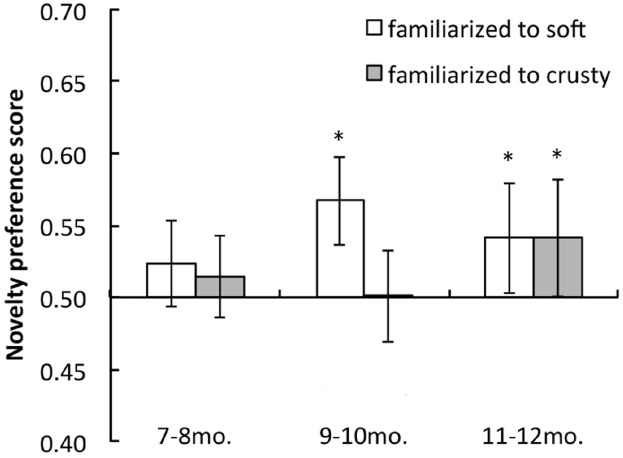
**Mean novelty preference scores for test trials under soft and crusty conditions for each age group in Experiment 1.** Error bars indicate 95% confidence intervals.

Figure 3 shows the mean novelty preference scores of each age group for soft and crusty conditions. The 7–8-month-old infants did not show a novelty preference under either condition. In contrast, the 9–10-month-old infants looked longer at the novel trials, though only under the soft condition; they did not show a novelty preference under the crusty condition. The 11–12-month-old infants looked longer at the novel trials under both conditions. Analyses involving two-tailed *t*-tests against a chance level of 0.5 revealed significant differences in looking times under the soft condition for 9–10- and 11–12- month age groups [age 7–8 months: *t*(17) = 1.73, *p* = 0.1014, *d* = 0.41; age 9–10 months: *t*(18) = 4.39, *p* = 0.0004, *d* = 1.01; age 11–12 months: *t*(18) = 2.10, *p* = 0.0489, *d* = 0.48]. The *t*-tests for the crusty conditions revealed significant differences in looking times only for the 11–12- month age group [age 7–8 months: *t*(17) = 1.03, *p* = 0.319, *d* = 0.24; age 9–10 months: *t*(18) = 0.07, *p* = 0.9449, *d* = 0.02; age 11–12 months: *t*(18) = 2.30, *p* = 0.0331, *d* = 0.53]. A two-way mixed analysis of variance (ANOVA) on age group (3) as a between-participants factor × stimulus condition (2) as a within-participants factor revealed a significant main effect of condition [*F*(1, 2) = 0.505, *p* = 0.0385] and marginally significant interactions between the two factors [*F*(2, 53) = 3.154, *p* = 0.0568]. However, the main effect of stimulus condition was not significant [*F*(2, 53) = 0.812, *p* = 0.4493]. *A posteriori* analyses (Ryan’s method) revealed significant differences in the preference scores of the 9–10-month-old infants.

These findings suggest that at least by 11 months of age, infants perceived differences in the hardness of the surfaces of penetrated objects based on changes in the velocity of penetrating objects. The surface texture of penetrated objects was identical under both the soft and crusty conditions. Therefore, the infants’ perception of the hardness of an object’s surface was influenced by velocity changes before and during penetration rather than by cues relating to the object’s texture. This would be the first evidence that infants have expectations regarding object attributes, such as the hardness of object surfaces, based on motion information.

However, an alternative explanation is that the crusty stimuli were so attractive that infants did not habituate to them. To rule out this possibility, we compared the total looking times for each stimulus during familiarization in 9–10-month-old infants (crusty = 32.91 s; soft = 31.43 s). A paired *t*-test revealed no differences in looking times between conditions [*t*(18) = 1.52, *p* = 0.1464, *d* = 0.28]. Therefore, the results cannot be explained by differences in stimuli attractiveness.

Even though the above explanation was ruled out, it still remains possible that the results of Experiment 1 are due not to infants’ ability to distinguish differences in the hardness of object surfaces but rather to their ability to distinguish differences in the velocity patterns of the stimuli. To rule out this possibility, in Experiment 2 we examined whether infants could discriminate between stimuli when only the movement of a stick was presented.

## Experiment 2

### Materials and Methods

#### Participants

This study involved 52 infants divided into three age groups: 7–8 months (*N* = 18, 10 males and eight females, *M* = 220.8 days, SD = 15.5 days), 9–10 months (*N* = 17, eight males and nine females, *M* = 283.3 days, SD = 16.0 days), and 11–12 months (*N* = 18, eight males and 10 females, *M* = 349.1 days, SD = 16.6 days). One additional infant participated in the experiment, but was excluded from the final sample due to excessive crying. Participants were recruited in the same manner as in Experiment 1. All parents of the participants provided informed consent. This study was approved by the Ethics Committee for Psychological Research, Niigata University, and was conducted according to the principles of the Declaration of Helsinki.

#### Apparatus

The apparatus was identical to that used in Experiment 1.

#### Materials

**FIGURE 4:**
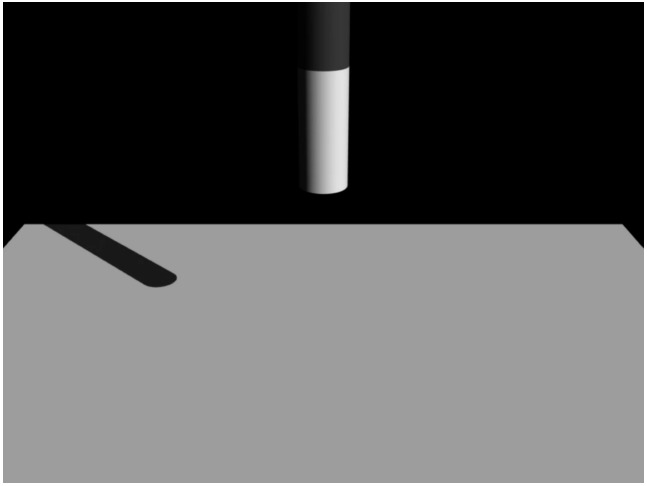
**Example of stimuli used in Experiment 2**.

The stimulus videos were identical to those used in Experiment 1, except that the yellow hemisphere was deleted from the videos in the current experiment (Figure 4).

#### Procedure

Infant were exposed to both of two experimental conditions, “crusty” and “soft.” Each experimental session consisted of four familiarization trials and two test trials. In the familiarization trials, each of the videos was presented for 9 s per trial. In subsequent test trials, two videos were presented one at a time for 9 s, as described in Experiment 1.

If the infants responded to differences in velocity changes in the stimuli rather than differences in object surface properties, they should still be able to discriminate between the test stimuli even in the absence of the hemispheric objects.

### Results and Discussion

**FIGURE 5:**
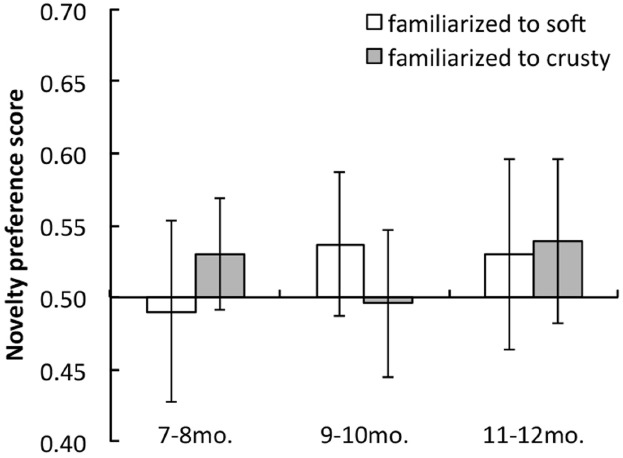
**Mean novelty preference scores for test trials under soft and crusty conditions for each age group in Experiment 2.** Error bars indicate 95% confidence intervals.

Figure 5 shows the mean novelty preference scores for each age group during the test trials. A statistical analysis of the looking times for infants in all age groups revealed no preference between stimuli [soft condition: age 7–8 months: *t*(17) = –0.53, *p* = 0.606, *d* = –0.12; age 9–10 months: *t*(16) = 0.52, *p* = 0.147, *d* = 0.37; and age 11–12 months: *t*(17) = 1.14, *p* = 0.269, *d* = 0.26; crusty condition: age 7–8 months: *t*(17) = 1.01, *p* = 0.328, *d* = 0.24; age 9–10 months: *t*(16) = –0.06, *p* = 0.866, *d* = –0.04; and age 11–12 months: *t*(17) = 1.28, *p* = 0.216, *d* = 0.30]. A two-way mixed ANOVA on age group (3) as a between-participants factor × stimulus condition (2) as a within-participants factor revealed that no main effects of age group [*F*(2, 50) = 0.433, *p* = 0.6512] or stimulus condition [*F*(1, 2) = 0.019, *p* = 0.8909], and no interaction between the two factors [*F*(2, 50) = 0.148, *p* = 0.2837]. These findings suggest that the infants did not identify the shapes of objects based on the movements of the stick in Experiment 1.

## General Discussion

We examined the perception of object surface hardness based on observation of penetration events in infants aged 7–12 months. The results of Experiment 1 suggested that at least by 11–12 months of age, infants can perceive differences in the hardness of the surfaces of penetrated objects based on changes in the velocity of penetrating objects. The results of Experiment 2 ruled out the possibility that the infants distinguished the stimuli based on differences in the movements of the penetrating object alone, rather than relying on attributions of the surface properties of the penetrated objects. These findings suggest that 11–12-month-old infants infer the hardness of object surfaces from motion information in the absence of tactile information.

The findings of Experiment 1 also demonstrated that 9–10-month-old infants showed a novelty preference for the crusty stimuli when they were familiarized with soft stimuli; however, they did not show a novelty preference for the soft stimuli when familiarized with the crusty stimuli. In contrast, 11–12-month-old infants showed a novelty preference under both conditions. These results imply that sensitivity to crusty surfaces may develop later than sensitivity to soft surfaces.

One possible interpretation of the difference in results between crusty and soft familiarization stimuli is that the crusty stimuli were more complex, making it more difficult for infants to judge the hardness of the object’s surface. According to Masuda et al. (2011), adults judged surfaces to be relatively hard when the moving stick either decelerated before penetration or accelerated during penetration (Crusty 1 and Crusty 2). Additionally, their study also investigated the perception of the “internal” hardness of objects. The results suggest that the inside of the hemisphere was perceived as relatively less hard when the average velocity of the stick increased during penetration (which is true for Crusty 1 stimuli) or when it decelerated during penetration (which is true for Crusty 2). These findings suggest that the crusty stimuli generate impressions pertaining to both the surface and the inside of an object, possibly leading to greater complexities for infants in judging an object’s hardness.

Previous research regarding material perception offers evidence that 5-month-old infants are able to distinguish liquids from solids using penetration and deformation cues (Hespos et al., 2009). The present study extended these findings of infants’ knowledge of the physical properties of non-solid objects. Infants began to infer the hardness of object surfaces from velocity changes before and during penetration between the ages of 9 and 12 months. These findings are also supported by a recent study of infants’ object exploration behavior with regard to surfaces with different material properties. Bourgeois et al. (2005) examined the manual exploration of objects involving surfaces of four different kinds of material (solid, discontinuous, flexible, and liquid) in 6-, 8-, and 10-month-old infants. The results indicated that even by 6 months of age, infants manipulated hard and soft objects in different ways, but that the manner of exploring an object’s surface varied across age groups. Only 10-month-old infants rubbed objects with a rigid surface more than those with other types of surfaces. In contrast, 6- and 8-month-old infants did not vary their exploration behaviors depending on the material property of the object’s surface. In Experiment 1 of the current study, infants had to infer hardness from interactions between a stick and the surface of a hemispheric object. A developmental study on haptic perception and object manipulation suggested that the emergence of a particular motor ability can be used to determine perceptual development (Bushnell and Boudreau, 1993). It is possible that tactile experiences of actions pertaining to objects and their surfaces are a necessary prerequisite for inferences of surface hardness from visual motion information, and such experiences accumulate gradually over the course of a child’s development.

In summary, our results demonstrate that at least by 11 months of age, infants perceive differences in the hardness of object surfaces based on observed velocity changes in penetration events. This is the first evidence showing that infants distinguish details of physical properties related to the material of objects.

It is still unknown how infants perceive different materials and qualities of objects. Previous psychophysical studies with adults have queried the kinds of visual parameters that contribute to humans’ perception of material properties. Several studies have suggested that the mechanism of humans’ perception of glossiness can be explained by a surprisingly simple mechanism (Motoyoshi et al., 2007). Recently, Yang et al. (2011) demonstrated that 8-month-old infants perceived differences between glossy and matte objects using surface representations. Future studies will undoubtedly contribute evidence of infants’ ability to distinguish the physical attributes of objects associated with various kinds of material texture.

### Conflict of Interest Statement

The authors declare that the research was conducted in the absence of any commercial or financial relationships that could be construed as a potential conflict of interest.
